# Well Performance Classification and Prediction: Deep Learning and Machine Learning Long Term Regression Experiments on Oil, Gas, and Water Production

**DOI:** 10.3390/s22145326

**Published:** 2022-07-16

**Authors:** Nehad M. Ibrahim, Ali A. Alharbi, Turki A. Alzahrani, Abdullah M. Abdulkarim, Ibrahim A. Alessa, Abdullah M. Hameed, Abdullaziz S. Albabtain, Deemah A. Alqahtani, Mohammad K. Alsawwaf, Abdullah A. Almuqhim

**Affiliations:** Department of Computer Science, College of Computer Science and Information Technology, Imam Abdulrahman Bin Faisal University, P.O. Box 1982, Dammam 31441, Saudi Arabia; 2180005979@iau.edu.sa (A.A.A.); 2180005928@iau.edu.sa (T.A.A.); 2180007107@iau.edu.sa (A.M.A.); 2180000853@iau.edu.sa (I.A.A.); 2180007122@iau.edu.sa (A.M.H.); 2170006078@iau.edu.sa (A.S.A.); daalqahtani@iau.edu.sa (D.A.A.); mkalsawwaf@iau.edu.sa (M.K.A.); aalmuqhim@iau.edu.sa (A.A.A.)

**Keywords:** oil, gas and water production prediction, machine learning, deep learning, random forest, recurrent neural network, artificial neural network, well performance production

## Abstract

In the oil and gas industries, predicting and classifying oil and gas production for hydrocarbon wells is difficult. Most oil and gas companies use reservoir simulation software to predict future oil and gas production and devise optimum field development plans. However, this process costs an immense number of resources and is time consuming. Each reservoir prediction experiment needs tens or hundreds of simulation runs, taking several hours or days to finish. In this paper, we attempt to overcome these issues by creating machine learning and deep learning models to expedite the process of forecasting oil and gas production. The dataset was provided by the leading oil producer, Saudi Aramco. Our approach reduced the time costs to a worst-case of a few minutes. Our study covered eight different ML and DL experiments and achieved its most outstanding R2 scores of 0.96 for XGBoost, 0.97 for ANN, and 0.98 for RNN over the other experiments.

## 1. Introduction

**Figure 1:**
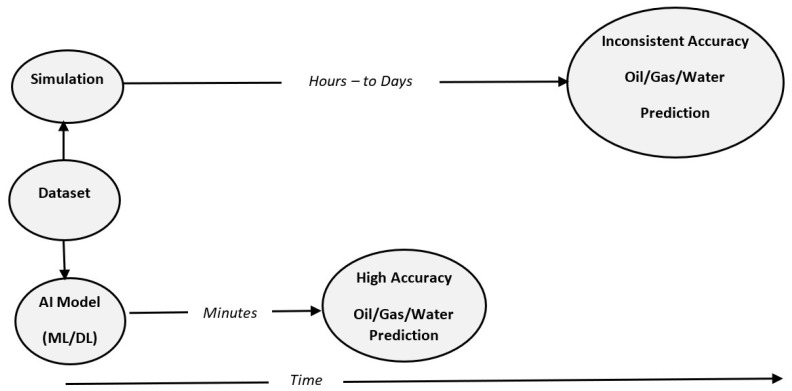
Abstract view of the problem and the proposed solution.

The ability to forecast oil wells’ production prior to drilling is a critical element in oil companies’ decision making. To do so, most oil companies, such as Saudi Aramco, use simulation. However, despite its accuracy, this method is time consuming due to the vast computational power required to perform such a task. What we offer in this paper is an alternative approach that will ease this step significantly and dramatically reduce the computational power needed. Figure 1 below shows a comparison between simulation and our proposed solution to this problem, which is making an AI model. Simulation processes require significant time, and result in an inconsistent accuracy, whereas our solution uses AI to provide accurate predictions promptly without compromising accuracy. The AI system will be able to make such predictions using ML and DL on the provided dataset and, with the trained system, the AI will be able to forecast the production of oil wells based on a few geological features.

**Figure 2:**
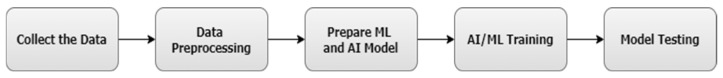
Flowchart for the research’s experiment, step by step.

We started our research by data collection. The data were provided by the well-known oil company Saudi Aramco. The data consist of a sample of simulation data representing the multiple reservoirs of oil wells with different numbers of wells. A total of five different reservoirs’ data were received as the dataset. These phases are illustrated in Figure 2. Data preprocessing, and the other steps shown in Figure 2, will be covered in detail in the upcoming sections.

## 2. Related Works

Many researchers in the oil industry have adopted machine learning and deep learning to predict the future with previous knowledge (supervised learning), such as reservoir history matching, forecasts for oil, gas, and water production, pattern recognition in well test analyses, and so on. In this section, we will cover work related to the application of machine learning in predicting oil, water, and gas production, as well as predicting the well pressure and well oil/gas/water ratio. The well production prediction process with the current methods used, such as simulation, takes a long time and is very costly in computational resources. Since our dataset is a continuous (time-series) problem type we had to look in-depth and explore the possibility of achieving high results with our dataset [1]. The current methods used create a bottleneck effect in building new wells. As technology has progressed, many different ML and DL techniques have been implemented to enhance production prediction performance. Moreover, this section was organized based on a preliminary search and understanding of the relevant machine learning studies dealing with well performance classification and prediction. Furthermore, the studies were thoroughly reviewed, and various ideas that are related to the main idea were gathered from different articles. Our next step was to break the literature review down into multiple areas of oil production relevant to our subject.

### 2.1. Oil Flowrate Prediction

A recent study related to artificial intelligence calculated the amount of oil flowing as a function of operational variables and choke size. The study used two different datasets for critical and subcritical flow: choke size, upstream pressure, temperature, gas/oil ratio, and water cut for critical flow. The subcritical dataset had the same dataset as the critical flow dataset, plus downstream pressure. The study then put a variety of AI techniques to the test, including artificial neural networks, fuzzy logic, and functional networks, as well as commonly used methods such as Gilbert correlation. When compared to current correlation approaches, which have a maximum correlation value of 0.30, the ANN system showed significant accuracy with a correlation coefficient of 0.89 [2].

A recent study discussed the oil flow rate through an orifice flow meter that was predicted using machine learning algorithms. Pressure, temperature, viscosity, square root of differential pressure, and oil-specific gravity were used as inputs from a dataset of 1037 data records. The ML methods used (adaptive neuro-fuzzy inference system (ANFIS), least squares support vector machine (LSSVM), radial basis function (RBF), multilayer perceptron (MLP), and gene-expression programming (GEP)) all achieved high levels of accuracy, with correlation coefficients ranging from 0.90 to 0.99 [3].

The authors described a state that tight gas fields are a substantial source of hydrocarbons for the energy industry. The process generates a large amount of information. As a result, data may be evaluated and predicted using machine learning methods., as well as being used to identify patterns between dependent and independent variables. The artificial neural network (ANN) and the generalized linear model (GLM) were employed in this study. The study’s goal is to figure out how well-planned new wells will recover. For production data, a dataset of 224 wells was examined. The results of these models were compared to the wells’ actual first gas production rate. Consequently, analysis found that a GLM model had a mean square error of 1.57 and an ANN model had a mean square error of 1.24. Furthermore, the performance index of the ANN model revealed that reservoir thickness was responsible for 36.5 percent of the initial gas output, followed by flowback rate (29%). As a result, when it came to estimating gas production and looking back, the ANN model outperformed the GLM model [4].

The authors described the predictions of the oil production flow rate in situations when direct measurement is difficult, which is a problem faced by petroleum engineers in several parts of the world. This study employs an artificial neural network approach to develop a new methodology for estimating oil flow rate in two-phase oil and gas flow through wellhead chokes. The artificial neural networks (ANNs) model is used to estimate oil flow rate as a function of the following parameters: choke upstream pressure, choke size, and the producing gas-to-oil ratio. The suggested model’s accuracy was compared to a number of well-known empirical correlations, and the results demonstrated that the new model’s predicted oil flow rates are very close to actual observed data. Furthermore, when compared to empirical correlations with more than three input parameters, this model takes less time to estimate the oil flow rate, with an accuracy rate of around 87% [5].

### 2.2. Well Production

A study has been proposed that discusses a hybrid model based on a mix of CNN and LSTM networks for time series forecasting of oil production. First, the CNN layer of the model is applied to the current time window, extracting the features, and then the LSTM is used to forecast the relationship between the time windows. The purpose of this research is to employ deep neural networks to construct a system that can accurately predict oil production based on well debt data. They arrive to the following conclusion: the CNN + LSTM model’s root mean squared logarithmic error (RMSLE) value is 0.186891 [1].

The authors described their work as not only developing a proxy model, but also testing it using field data from 1239 horizontal wells. The following findings were obtained as a result of this research. Initially, the exploratory data analysis (EDA) technique was utilized to investigate the datasets, which included outlier analysis, categorical and numerical variable analysis, and correlation analysis. Through EDA, 1150 wells were used as training data from 1239 wells. Second, principal component analysis (PCA) was utilized to reduce the input variable’s size. Finally, VIA used the RF, GBM, and XGBoost algorithms to identify independent variables that had a substantial impact on cumulative gas production over a 12 month (CGP12) forecast. The average relevance ranking of the independent variables was obtained using this method. Finally, hyperparameter sensitivity analysis is used to construct DNN models that are more predictive [6].

A recent study that discussed the use of RNN-LSTM to estimate oil, gas, and water outputs of wells based on injection patterns in a time-series manner was investigated by the author. The RNN-LSTM model was able to estimate oil, water, and gas production with a first-year accuracy of over 90% and production values for up to 5 years with a 73.63 percent accuracy [7].

The authors described their work by proposing an automated method for assisting technical teams in increasing data quality in production data analysis using machine learning techniques, improving reliable production forecasting, reducing operating costs, and optimizing drilling schedules. Reservoir pressure, water cut, wellhead pressure, choke size, and rate were all used as input features in this study. The following machine learning approaches were used to efficiently eliminate outliers and noisy data. The K-means clustering algorithm, for example, is used to find outliers or abnormalities. Second, support vector regression (SVR) is a powerful tool for removing noise from data during the analysis stage. The adoption of the aforementioned strategies can improve the accuracy of production forecasting [8].

The research was conducted to describe the advocates’ use of artificial neural networks to forecast oil, water, and gas output in water-injected reservoirs. To train the ANN model, the researchers employed the Bayesian regularization approach. A coefficient of determination of more than 0.9 was achieved by the ANN model. The researchers also discovered that the delay in the time-step term is a significant element that can improve the model’s prediction because other models ignore it [9].

A study has been proposed that discusses the notion that many elements, including geology and completion, play a role in gas output. We can create a production prediction model and determine the most important element affecting production using machine learning approaches. The Duvernay formation was targeted with 159 horizontal wells. Then, grey-connection analysis and Pearson correlation were used to find the essential factors. Finally, multiple linear regression (MLR), support vector regression (SVR), and Gaussian process regression were used to create three statistical models (GPR). Cumulative oil and gas production were forecast for the first six months of production [10].

A study has been proposed that discusses a hybrid model using the attention mechanism and combining it with the convolutional neural network (CNN) and the long short-term memory neural network, thus creating (attention-CNN-LSTM). The data used for this study are collective daily data for two wells in an oilfield in southern China. T1 well data have been collected over 23 years, each day is a different record, and for T2 well data it was for only 17.5 years. Other algorithms have been tested as well, such as support vector regression (SVR), back-propagation neural network (BP), regular CNN LSTM, and more. However, according to three different comparison measures, attention-CNN-LSTM appears to have the lowest score uncontested which means it provides the highest accuracy among all other models with an RMSE (root mean square error) score of 0.315 and 0.402, an MAE score of 0.218 and 0.303, and a MAPE (mean absolute percentage error) score of 0.008 and 0.005 for T1 and T2 wells, respectively [11].

A recent study discusses the proposed methodology for estimating oil production from a single well. They used the LSTM method. However, the model itself has been modified and manipulated to achieve a better result. The training speed and generalization capabilities of the model can be increased by using several optimization strategies, such as the batch normalization layer, which improves prediction accuracy. The used dataset contains 1275 wells, from January 1973 to May 1995. Moreover, the used dataset contained 43 features. The author also introduced a data-labeling method depending on the different water cut stages. With four phases, each well must belong to at least one. The tool also offers other labeling methods, such as well type. After the data preprocessing by the tool, they selected 65 wells from the dataset, which has been divided into 90/10, 1409 samples for the training. The remaining 157 samples were considered for the test set. As well as the LSTM, the author used RF and SVM to compare them. The used metrics are RMSE, MAS, and R2. For the RMSE, LSTM has the lowest value of 383.33 which indicates a better performance. Furthermore, in MAE, LSTM has the lowest value of 285.34. the R2 of LSTM was the highest of 0.786 of all the methods [12].

According to the article, the three ANNs architectures were developed based on simulation data from 370 reservoir simulations, hydraulic fracture design parameters, and fracture network properties, including fracture spacing and fracture conductivity. These parameters significantly affect shale gas production. Based on another set of 92 simulations, the testing results ensured a high correlation between input and objective functions, with an R2 > 0.86. In addition, good agreement was found between measured and predicted cumulative gas production over 1, 5, 10, and 15 years, with an R2 > 0.94, and with error rates under 15%. Using the peak production rate can improve prediction accuracy for wells that have been producing for a short time [13].

### 2.3. Well Production Enhancement Prediction

A study has been proposed that discusses a unique deep recurrent neural network that was constructed to be used by petroleum engineers to analyze the features of producing oil wells. In addition, this paper used the deep structure of a GRU recurrent neural network that serves as the foundation for the model and compares the most popular time-series models. The paper aims to analyze and compare the strength and precision of the layered DGRU model to the most popular time-series models. After pre-processing the dataset, they end up with a collection comprising 21,000 observations of US oil production data spanning 90 years. The entire collection of the dataset is separated into three subgroups: training 40%, validation 20%, and testing 20%. Furthermore, this approach may be used to measure the trained model’s performance as well as the stacked deep GRU model’s performance using the testing dataset (unknown new data). Moreover, the DGRU model compared accuracy and performance to different time-series models. The models included in this study are multi-RNN, single GRU, Multi-GRU/ANN, Multi LSTM. The overall accuracy performance results, using R2 measurement with 10 steps prediction, were:

- Multi-RNN: 5.523%
- Single GRU: 55.078%
- Multi-GRU/ANN: 72%
- Multi LSTM: 51%
- Stacked DGR: 70%

They found that the model’s construction is substantially simpler than that of LSTM and RNN. Because of its low-complexity structure and ability to handle long-interval time-series datasets, our DGRU model outperforms other conventional models. As a result, our DGRU model may be used for the long-term dependencies of a complicated time series dataset [14].

### 2.4. Pressure Gradient Prediction

Research was conducted to describe the use of a multilayer feed-forward ANN and was able to predict the horizontal oil–water flow pressure gradient using five inputs which are: oil superficial velocity, water superficial velocity, pipe diameter, pipe roughness, and oil viscosity. Using 765 experimental data, the data were divided into three sections: training (60%), validation (20%), and testing (20%). The ANN model had an APE of 0.30 percent, an AAPE of 2.9 percent, and an SD of 7.6 percent, which is extraordinarily low when compared to the other approaches studied (two-fluid model, homogeneous model, and correlation), which had an average AAPE of 22.88 percent [15].

The authors described their work by proposing machine learning algorithms that were carried out. The pressure gradient in a liquid–liquid flow was determined using six different approaches: support vector machine (SVM), Gaussian process (GP), random forest (RF), artificial neural network (ANN), k-nearest neighbor (kNN), and a fusion model are among the algorithms used. Seven predictor values were chosen as the best collection of predictors with the fewest mistakes using SVM. Oil and water velocities, FP, input diameter, oil and water density, and oil viscosity are the seven predictors. In comparison to other models, the GP model had the highest prediction accuracy, with the exception of the ML-fusion model, which had a (*p* < 0.05) [16].

### 2.5. Fault Prediction

Research was conducted to describe the preliminary construction of a machine learning (ML) model for early defect prediction of a centrifugal pump in the oil and gas industry that is simple and easy to deploy. The selected machinery’s process and equipment sensors were used to examine real-time historical data. To train the model, raw sensor data, mostly from temperature, pressure, and vibration probes, were denoised, pre-processed, and sequentially coded. This paper compares two algorithms: support vector machine (SVM) and multilayer perceptron (MLP). The SVM algorithm noted a high accuracy value of 98.1%. The MLP algorithm recorded an overall accuracy of 98.2%, which was slightly higher than the SVM algorithm. This study’s primary purpose is not to create high-accuracy ML models but rather to demonstrate that it is possible to have good forecasts with a simple and intuitive ML algorithm. Overall, the results suggest that the proposed algorithms perform well in identifying the health status of the monitored machine, providing good overall classification performance [17].

A study has been proposed that discusses a novel hybrid LSTM–SAE learning method that will be used to overcome the weaknesses of RNN training and the use of a single technique individually, as well as increase the fault detection accuracy. The DCS of the electrical generator provides all of the vibration signals. Before being fed to the proposed DL framework, approximately 2000 samples were separated into a training dataset (80%) and a testing dataset (20%). These samples are divided into two categories: faulty and non-faulty training-and-testing datasets. The sample data were chosen and used to train and test RNN-LSTM for usage in the proposed DL framework’s fault detection phase. This study planned to purpose the following primary contributions to the current work:

- Based on the RNN-LSTM, SAE, and particle swarm optimization (PSO) approaches, a novel DL fault detection with a simple and effective framework is created to balance the three steps of parameter optimization, fault feature extraction, and fault detection.
- A novel hybrid mathematical approach can improve learning ability by addressing RNN training limitations such as decaying error, deficit, gradient vanishing, and backflow.
- The DL framework provides strong autonomous deep learning for unlabeled data, allowing the proposed DL approach to not only adapt the relevant features, but also to realize patterns without saving the prior sequence inputs.
- The suggested deep learning framework contributes to the field of electrical gas generator defect detection, which could be valuable for future industrial deep learning applications, particularly in dangerous environments.

Due to benefits in the feature extraction and fault detection stages, the proposed framework outperforms competing approaches, with a detection accuracy of 99.67 percent. Furthermore, the results reveal that the proposed deep learning system can recognize faults in industrial data without labels. This can professionally assist data engineers in automatically extracting features, and avoid reliance on human experience based on the unsurprised defect detection approach. Moreover, we can see according to this study that the accuracy of the proposed framework is the highest when compared to other approaches, which are RNN, and ANN with the accuracy of (69.0%), and (49.5%), respectively. When compared to existing approaches, the suggested DL framework has a higher time efficiency with a faster detection time (0.17 s) [18].

### 2.6. Bottom-Hole Pressure Prediction

In order to determine the pressure drop in wells, it is important to calculate the bottom hole pressure (BHP). The author used ANN with a hybrid genetic algorithm and particle swarm optimization (HGAPSO) to predict the BHP. There are nine inputs used: oil flow rate, gas flow rate, water flow rate, oil API, depth, tubing diameter, temperature at the surface, wellhead pressure, and bottom-hole temperature [19]. Testing this model on a population of size 150 has yielded results with a maximum error of 10%. Although other methods have been tested, such as GA-ANN and PSO-ANN, the hybrid method HGAPSO-ANN has shown the greatest success rate. Another method to calculate BHP is demonstrated in the article [20]. The author used gradient tree boosting (GTB) and extreme learning machine (ELM). The dataset used consisted of eight parameters: wellhead pressure, oil flow rate, water flow rate, gas flow rate, average deviation, average angle, measured depth, and true vertical depth. The use of these two methods has yielded a mean relative error lower than 4%. Furthermore, a recent study discusses the ANN, KNN, and random forest to predict the flowing bottom hole pressure (FBHP). Using these methods with the following nine inputs: flowing oil rate, flowing gas rate, flowing water rate, production tubing internal diameter, well perforation depth, oil gravity, surface temperature, well bottom hole temperature, and wellhead pressure has yielded an error rate of 2.5%, 3.6%, and 4% for ANN, random forest, and KNN, respectively [21].

### 2.7. Reservoir Characterization

Using machine learning and dynamic production data, research was conducted to describe and suggest an alternate technique for predicting vertical heterogeneity in reservoirs. They used numerical simulation techniques to gather dynamic production data from a variety of heterogeneous reservoir conditions. In comparison to traditional approaches, the machine learning model demonstrated outstanding predictive accuracy on vertical permeability, with an RMSE of 12.71 MD, effectively estimating the permeability of the whole reservoir rather than a specific point. Eventually, the trained machine learning models can accurately invert reservoir permeability. The total AARD of the prediction result generated by the CNN technique was controlled at 11.51 percent based on model validation, which was lower than the BP and LSTM network in calculating error. Simultaneously, the prediction time of the three neural networks was extremely fast, at around 1 s. As a result of the complete study of accuracy and prediction time, CNN may be chosen as the best model. With a derivation of less than 10%, the machine learning technique can forecast permeability contrast with excellent accuracy in a variety of heterogeneous reservoir conditions [22].

A study has been proposed that discusses, without any well-specific calibration and/or other inadequacies of existing approaches, the use of ANN to forecast gas hydrate saturation using a dataset consisting of porosity, bulk density, and compressional wave (P-wave) velocity well logs. According to three independent studies, the proposed method has an accuracy of 84 percent in estimating gas hydrate saturation, which is higher than the 75 percent accuracy of the currently utilized methodologies: seismic and electrical resistivity approaches [23].

A recent study that discusses using deep mutual information classifiers and a multi-feature-extraction approach proposes a novel integrated well-testing interpretation model (MFE-DMIC). The data in this report come from Huabei Oilfield’s well-testing platform. Special lithology, structural cracks, and powerful edge water characterize the reservoirs. A total of 4004 stage samples of oil testing data, and the accompanying operating stages, are considered in the study. The training set to testing set ratio was 6:4. Therefore, they identified 2402 stage samples as the training set and 1602 stage samples as the testing set at random. They started by employing four typical feature-extraction methods to retrieve the basic characteristics. They then employed a deep belief network to eliminate feature redundancy before achieving feature purification using the maximum information coefficient method. Finally, they used a hybrid particle-swarm optimization–support vector machine classification method to calculate the interpretation findings. This will reduce the efforts of oil analysts and allow for accurate sample labeling to be predicted. They achieved 98.18% stage classification accuracy [24].

The authors described their work by proposing a new well-testing stage classification method based on a deep vector learning model (DVLM) which is a combination of multi-feature extraction, deep learning, and feature vector mapping. The data used in this article come from the Huabei Oilfield’s well-testing platform. Special lithology, structural cracks, and forceful edge water are all features of the reservoirs. The study takes into account a total of 4004 stage samples of oil testing data as well as the associated operation stages. They randomly categorized 2402 stage samples as the training set and 1602 stage samples as the testing set in a 6:4 ratio. They began by extracting the data’s basic features using four standard feature extraction methods. They then used a five-layer deep belief network with the mutual information coefficient approach to extract more features and clean the data. Lastly, the data are classified using the optimized learning vector quantization classifier, so the predicted tags are output. This will decrease the efforts of oil analysts and help to predict accurate sample labeling, with a total result of 98.065% stage classification accuracy [25].A study has been proposed that discusses anticipated deliverability. Researchers used a dataset from the US Energy Information Administration (EIA), which had 864, 432, and 216 records for the years 2017 to 2020, respectively. Total field capacity, base gas, working gas, and capacity are all handled in the data record. Support vector machine (SVM), artificial neural network (ANN), and random forest (RF) machine learning (ML) methods were used in this study to predict the deliverability (dependent variable) of UNGS in salt caves, as measured by (Mcf/day), which stands for million cubic feet per day. The result demonstrates that the RF model outperforms the ANN and SVM models on all data samples [26].

### 2.8. Related Work Summary

To conclude, machine learning is incapable of handling non-linear complex problems. On the other hand, deep learning is most optimal for complex non-linear problems; therefore, our system will use deep learning to predict the oil/water/gas production rates as a first step. After completing the first step we look to expand the system to offer more deep-learning-supported solutions to predicting vital parameters in oil extraction such as: predicting oil/water ratio, predicting the well pressure, and predicting the flow rate. We aim to explore the use of RNN, as it is perfect for our problem type (time-series). Other methods, such as ANN and CNN, are not as viable; CNN excels at image recognition and can be used with RNN to improve results of the RNN module and, although ANN is viable for the prementioned task, it is less efficient in a time-series problem. However, more exploration with different methods will be needed to increase the efficiency of the system.

## 3. Methods and Materials

For our methods, we used eight different methods to compare them and try to find the best out of them. We will briefly explain each method in general and the formulas as well. Moreover, we will go through the dataset and the materials we used that helped us build the AI model.

### 3.1. Dataset

The five datasets we received are samples from Saudi Aramco of five well reservoirs with a different number of surrounding wells to predict oil/gas/water productions over almost three years. We combined all five datasets into one custom dataset in order to let the models learn the effect number of surrounding wells had on productions. The final combined custom dataset had 12 features and 280 dependent variables and a total of 1968 data points to work with. We can see each column and the description for it in Table 1

### 3.2. Tools

For our experiment we used Anaconda Spyder and Google Collaboration online compilers as environments to program in Python. This section will describe each library we used and why we used it. Sklearn is the main machine learning library. It includes most of the classification, regression, and clustering techniques, along with dataset splitting and fitting into multiple ML models. Tensorflow and Keras are libraries that facilitate the programming process for DL models and allows parameter tuning along with number of neurons and hidden layers. NumPy is a library that allows the use of arrays in Python. The Pandas library allows importing and splitting of datasets, and the Matplotlib library provides visual representations of the datasets. It can provide static, interactive, or animated graphs.

### 3.3. Methods

#### 3.3.1. MLR

As we know that regression is a way to predict the nature of the relationship between different variables, we use multiple linear regression (MLR) to find the relationships of a dependent variable with numerous independent or predictive factors. In MLR we can predict the dependent variable by two or more variables. As a result, MLR examines the correlation between numerous independent variables and the dependent variable. MLR can also help us know the value of the dependent variable at a specific independent variable value, we can use this information to acquire any dependent variable at any given point [27], the general for multiple regression is shown in Equation (1):

(1) $$Y=a+b_{1}\left(x_{1}\right)+b_{2}\left(x_{2}\right)+b_{3}\left(x_{3}\right)+\cdots +b_{n}\left(x_{n}\right)+\in$$

where *Y* refers to the dependent variable, *x*_1_, *x*_2_, *x*_3_, …, *x_n_* are the independent variables, *b*_1_, *b*_2_, *b*_3_, …, *b_n_* are the regression coefficients, a is the constant, and ε if the error. The regression coefficients in this equation stand for the independent contributions made by each independent variable to the forecasting of the dependent variable. Given the independent variables (*X*), the regression line expresses the most accurate forecast of the dependent variable (*Y*). There is usually a significant variance of the observed points around the fitted regression line since nature is rarely entirely predictable. The term “residual value” refers to the departure of a certain point from the regression line. Model fit is measured using R square, also called the coefficient of determination, which is equal to 1 minus the residual variability ratio.

#### 3.3.2. PLR

Polynomial linear regression (PLR) is one of the types of linear regression, and it can solve the problem when the relation between the variables is non-linear. It can help us determine the independent and dependent variables’ curvilinear relationship. PLR works by fitting the data into the model as a polynomial of the nth degree. We use PLR when the linear regression cannot capture the point in the data and fails to describe the best result. This will help us because the relation in our dataset is not linear [28], the polynomial linear regression is shown in Equation (2):

(2) $$Y=a_{0}+a_{1}\left(x_{1}\right)+a_{2}{\left(x_{1}\right)}^{2}+\ldots +a_{n}{\left(x_{1}\right)}^{n}$$

One downside for PLR is that, as the polynomial degree increases, the time cost of the model also increases. A very high-degree PLR model can provide highly accurate results but, in some cases, may cause the models to overfit. A low-degree model may cause the model to underfit as it will not learn or extract all the features in the dataset. We can solve this problem by using the Bayes information criterion (BIC), which is an external algorithm that can help us in determining the best degree for the PLR model.

#### 3.3.3. SVR

SVM is a well-known machine learning method that is used in classification for predicting one output. However, in our experiment, we will be using SVR which is an extension of SVM that allows it to handle regression problems, as it was limited to classification. SVR uses a function *f*(*x*) to transform a low-dimensional non-linear dataset into a high-dimensional linear problem in feature space by mapping the data with the function [29,30]. We still faced a problem as SVM/SVR both predict only one output whereas our experiment required us to predict 35 different outputs for oil, gas, and water predictions each collecting around three years of predictions. The use of a multi-output regressor is necessary, as it allows the model to act like other multi-target regression methods. The regression model of SVR can be written as follows, after numerous processes to the regression model. The Equation (3) of SVR is:

(3) $$f\left(x\right)=\overset{m}{\underset{i=1}{\sum }}\left(a_{i}^{*}-a_{i}\right)\;\delta \left(x_{i},x_{j}\right)+b$$

where δ(*x*) refers to kernel functions which differ depending on the use of the model. According to our experiment we used a radial basis function (RBF).

#### 3.3.4. DTR

Considering that our dataset is regression data, we decided to implement decision-tree regression using Python. A few reasons for choosing this ML method are how simple it is to implement and validate. Moreover, the computational cost to using the tree is relatively low, being [31], as shown in Equation (4).

(4) $$O(\log n_{training\;samples})$$

Moreover, the regression tree is somewhat more complex than the classification tree. The decision tree regression optimally splits up the data into sections called leaves by using the value of the threshold to answer the following questions:

- Does performing the split increase the amount of information we have about our dataset?
- Does it add some value to the approach we would like to group our data points (information entropy)?

The algorithm does stop when it has reached a certain minimal amount of accepted information. Then the following information is used to create a decision tree according to the data that come from each split with the parameters; lastly, the algorithm takes the average of the terminal leaves points (*Y*) within each split, so when a new point comes (*x*_1_, *x*_2_, ...) the model predicts its results with the average of (*Y*) value with the following Equation (5):

(5) $$\bar{Y}=\frac{1}{n}\underset{i\in n}{\sum }\left(Y_{i}\right)$$

Let *n* be the number of samples. This algorithm improves accuracy by splitting the points, then taking the average of all points in each split, which generally results in a much higher prediction outcome for the new element [32].

#### 3.3.5. RFR

Random forest regression (RFR) is a regression technique based on machine learning techniques. Bagging and random subspace approaches are at the core of it. Bagging is used to generate a variety of decision trees, which are then ensembled to obtain the overall prediction. To train the learner trees, several independent bootstrap samples were constructed from the primary training data. Each bootstrap sample Db is made up of N instances drawn in D. The general Equation (6) for random forest regression prediction is:

(6) $$RFR\;prediction=\frac{1}{K}\sum_{k=1}^{K}h_{k}\left(x\right)$$

Db is approximately 2/3 the size of D and does not contain any duplicate instances. For bootstrap samples with input vector *x*, a total of *K* number of independent decision trees are created using the DTR method discussed above. Moreover, the replacement of examples is possible while contracting bootstrap samples. High variance and low bias characterize the regression trees. In regression tasks, the random forest prediction is generated using the mean prediction of *K* regression trees, *h_k_*(*x*) [33].

#### 3.3.6. XGBoost

We can simply define XGBoost as a set of decision trees constructed sequentially. In XGBoost, weights are very significant. All of the independent variables are given weights, which are subsequently fed into the decision tree, and which may be used to solve issues including regression and classification. The weight of factors that the tree predicted incorrectly is increased, and these variables are fed into the second decision tree. Individual classifiers are then combined to form a more powerful and precise model. XGBoost was created with careful consideration of both system optimization and machine learning techniques. The purpose of this model is to push machines to their boundaries in terms of computing in order to create a flexible, portable, and accurate model.

XGBoost is a kind of gradient-boosted decision tree (GBM) that is optimized for both performance and speed. XGBoost has many features such as gradient tree boosting. In Euclidean space, standard optimization methods cannot enhance the tree ensemble of classifiers. The model is instead taught in an incremental approach. Moreover, regularized learning aids in smoothing the final learned weights, preventing over-fitting from the dataset size, etc. The regularized target will favor models that use basic and predictive functions. Friedman proposed the very first approach, reduction, and column dimension reduction, which are two more approaches used to avoid overfitting in addition to the regularized aim. After each stage of tree boosting, shrinkage adjusts recently added weights by a ratio. Reduction, like a learning rate in stochastic optimization, decreases each tree’s influence while allowing future trees to enhance the model. When the number of features in the training set is smaller than the number of observations in the training set, or if the dataset exclusively contains numeric features, XGBoost is utilized. XGBoost works in a similar way to a decision tree in that it creates a specific number of trees depending on the issues, but it does it one by one, with each following tree using the knowledge obtained by the previous tree to enhance it [34]. To put it another way, any new tree will correct the mistakes caused by the prior tree. XGBoost uses the following Equation (7): [34]
(7)
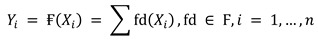


#### 3.3.7. ANN

Artificial neural network (ANN) is a method that simulates how the biological brain works. It is a deep learning method that consists of three types of layers: input layer, hidden layers (number varies between different models), and output layer. The input layer receives the inputs from the dataset, assigns weight values to them, and passes them to the hidden layers. An activation function is assigned to each neuron and a bias variable is added to the data. The output of the neuron is then sent to the next neuron in the next hidden layer. Each hidden layer can use different activation functions. At the end, all outputs are collected into the output layer where the last process on data is done to predict where the data belong. For our experiment we used 35 output neurons for each experiment since we are trying to obtain 35 different outputs, predicting the productions of oil, gas, and water productions over three years. Basically, ANN uses a collection of interconnected neurons through multiple layers that receive inputs *x_i_* with weights value related to each input *w_ij_* and a bias value, which allows shifting the activation function by adding a constant with its related weight as we can see Equation (8):

(8) $$N_{j}=\overset{n}{\underset{i=1}{\sum }}w_{ij}\left(x_{i}\right)+b_{j}$$

where *N_j_* represents the set of data coming from the *j*-th neuron. The neuron output is computed by the various activation functions. The output of the *j*-th neuron and the activation function can thus be represented as in Equation (9):

(9) $$Output_{j}=f\left(N_{j}\right)$$

where *f* varies among the choice of activation function, which is based on the type of problem the model is built to answer [27].

#### 3.3.8. RNN

RNN is a type of artificial neural network that processes sequential data to recognize patterns and predicts the final outcome. A similar calculation takes place for each element of a sequence, and the following output is based on a preceding calculation of the result. As part of its internal memory, RNN can remember or memorize the information of the input it received, which helps it to gain context and predict the next step. In order to anticipate the output of a layer, RNN saves the output of that layer and feeds it back into the input, and that is how RNN works. Moreover, it is one of the most powerful models when it comes to recognizing sequences of words and paragraphs, as well as predicting time series problems [35].

## 4. AI Model

**Figure 3:**
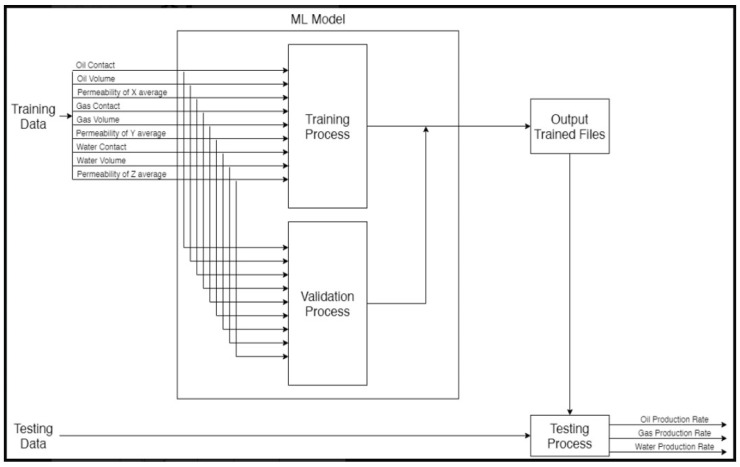
AI Model.

Figure 3 shows the model that we are building. We divided the dataset between the training and the test to 80% and 20% respectively. The AI model will use 80% of the dataset for training and 20% for testing. After finishing the training process, the evaluation process comes to measure the model. Hence, when the validation process takes place, it will use the split to ensure that the model is achieving accurate results. After finishing these two processes, we will be at the output trained files. Here we will use 20% of the remaining dataset to verify the result we obtained from the model. 

## 5. Result and Analysis

For comparing the results of our eight experiments, we used the R2 (correlation coefficient) score to determine which of the models had the best performance. Later, we applied other testing measures such as MAE (mean absolute error), MSE (mean squared error), and RMSE (root mean squared error) to the best-performing models. Table 2 shows all the used parameters in the study for all the eight models we experimented on. These parameters were optimized by applying a grid search algorithm that compared the results of the models for multiple different combinations. These parameters have resulted in the R2 scores for each model as the next sections will show.

### 5.1. MLR

**Figure 4:**
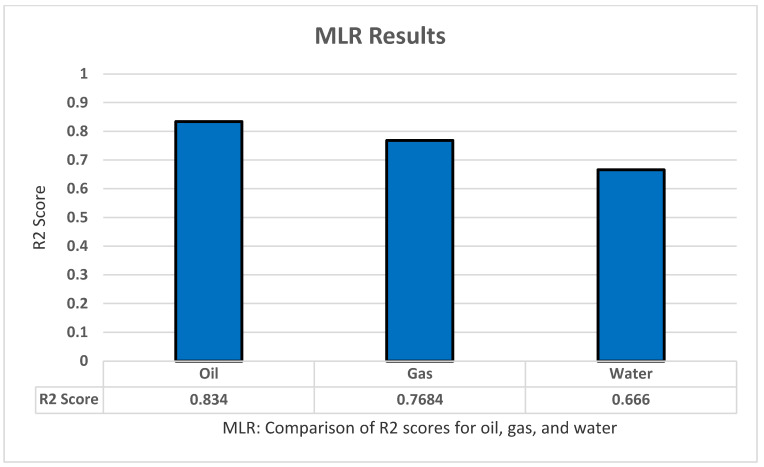
MLR’s R2 score results.

For MLR testing we used Sklearn LinearRegression to implement the model in Python. The model has a few parameters such as normalize and fit_intercept. However, those parameters are irrelevant to our model, as well as the fact that the data received are ideal state and the fact that normalization does not affect linear regression. Therefore, no further modification to the model or the data was implemented. For the train/test split we performed for the dataset is 80% for training, and 20% for testing and this is true for all methods. The model was used to predict the oil production, all productions (oil, water, and gas), as well as all dependent variables (all production + oil/water/gas ratio). Using k-fold cross-validation of five folds and five repeats, the model produced adequate results for the most part, with R2 of 0.834 for oil production, 0.7684 for gas production, and 0.6666 for water production, and a bad result of −0.02 for all dependent variables as shown in Figure 4.

### 5.2. PLR

**Figure 5:**
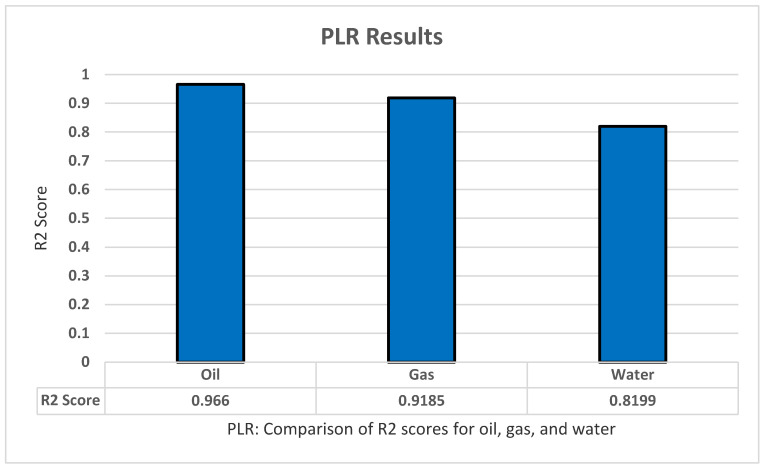
PLR’s R2 score results.

We tested polynomial regression using the LinearRegression model from the Sklearn library. As for MLR there is no modification to the model or the data. However, we tested the model using different degrees (from two to eight). As with MLR, three tests were conducted on oil, all production, and all dependent variables. The model performed better with an R2 score of 0.966 for oil production, 0.9185 for gas production, and 0.8199 for water production, all by using the degree of four in the experiment as shown in Figure 5.

### 5.3. SVR

**Figure 6:**
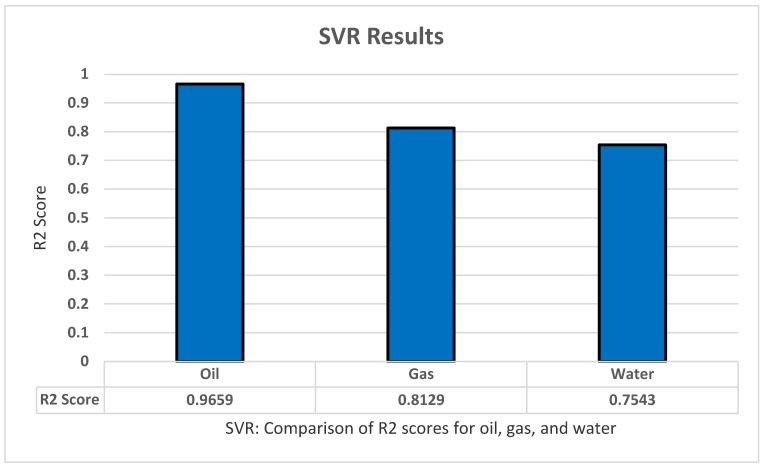
SVR’s R2 score results.

The SVR model experiment resulted in some good findings. However, these results were the lowest among all the other models. A repeated k-fold algorithm of five splits and five repeats was used in a grid search algorithm to tune the parameters of SVR. The parameters we found to achieve high results are: kernel with ‘Rbf’, Gamma ‘scale’, C ‘475’, Epsilon ‘0.01’, Max_iter ‘−0.1’, and Tol ‘0.1’. The model achieved great results with an R2 score of 0.9659 for oil productions, a score of 0.8129 for gas productions, and a score of 0.7543 for gas productions. An overall R2 score of 0.7401 was found for all dependent variables. All these results have been achieved after standardizing the data for the model as shown in Figure 6.

### 5.4. DTR

**Figure 7:**
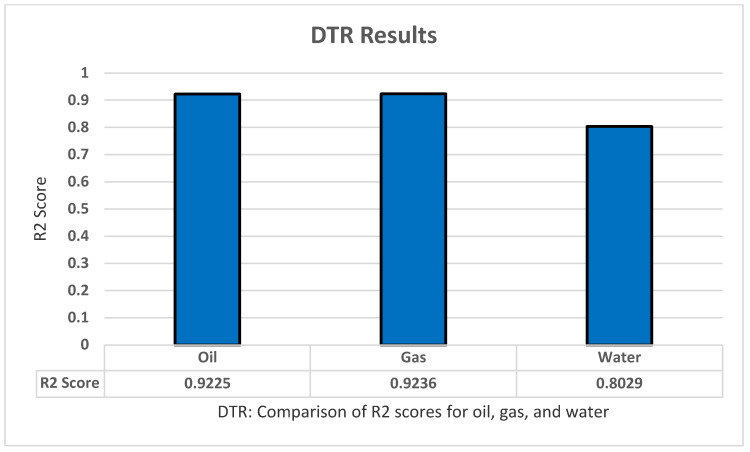
DTR’s R2 score results.

For testing the decision tree regression model, we apply sklearn.tree to implement the model using Python. We also implemented standardization and normalization for the dataset. Moreover, we applied the grid search technique to optimize the model parameters and increase the model accuracy as much as possible. We found that these parameters produce the highest results: Criterion ‘absolute_error’, Max_depth ‘6’, Max_features ‘auto’. Furthermore, the decision tree regression model has shown the result using R2 with K-fold cross-validation to predict all of the oil production, all productions (oil, water, and gas), as well as all dependent variables (all production and oil/water/gas ratio) with five folds, five repeats, and with random_state = 1 for standardization. The highest results were for the pure with R2 of 0.9225 for oil, 0.88 for all production, and 0.87 for all dependent variables as shown in Figure 7.

### 5.5. RFR

**Figure 8:**
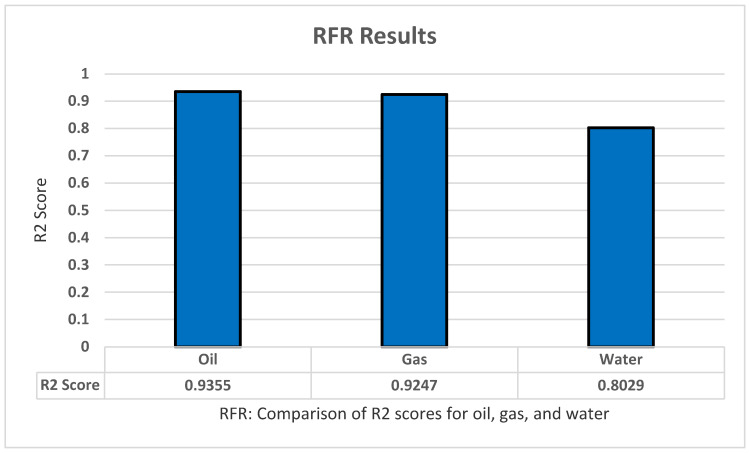
RFR’s R2 score results.

For testing the random forest regression model, we apply sklearn.tree to implement the model using Python. We also implemented standardization and normalization for the dataset. Moreover, we applied the grid search technique to optimize the model parameters and increase the model accuracy as much as possible. With this set of features, we achieved the highest results with: Criterion ‘squared_error’, n_estemators ‘100’, and Max_features ‘auto’. Furthermore, the random forest regression model has shown the result using R2 with K-fold cross-validation to predict all of the oil production, all productions (oil, water, and gas), as well as all dependent variables (all production and oil/water/gas ratio) with five folds, five repeats, and with random_state = 1 for standardization. The highest results in R2 were 0.9355 for oil, 0.9247 for gas, and 0.8029 for water production, as shown in Figure 8.

### 5.6. XGBoost

**Figure 9:**
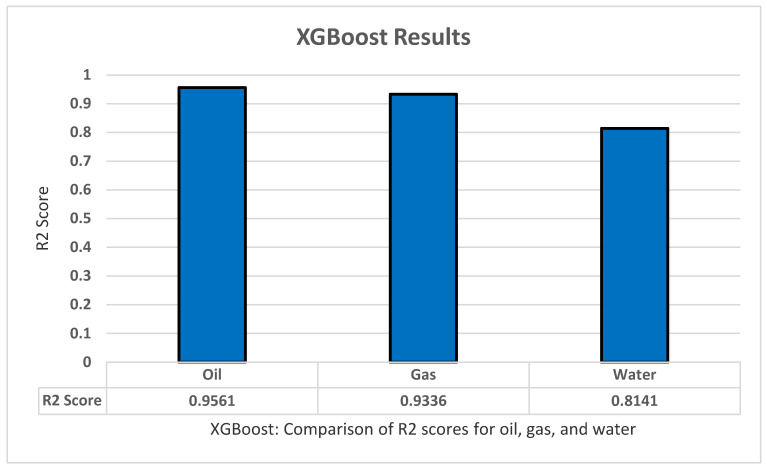
XGBoost’s R2 score results.

As for the XGBoost training, we used the same procedures as the previous methods. We again used k-fold cross-validation of five folds and five repeats, grid search, and fitting of the model itself. We used the XGBoost library to implement it easily, and we also implemented standardization and normalization for the data. XGBoost contains many parameters that can affect the result so, by using the gird search on the method several times in some parameters, we then took the best parameters and their values: Max_depth ‘2’, Learning Rate ‘0.4’, booster ‘gnlinear’, Gamma ‘0’. The model was used to predict the oil production, all productions (oil, water, and gas), all dependent variables (all production + oil/water/gas ratio), as well as the oil, water, and gas separately. XGBoost managed to achieve an R2 score of 0.9561 for the oil, 0.9336 for all gas, and lastly 0.8141 for water production as shown in Figure 9.

### 5.7. ANN

**Figure 10:**
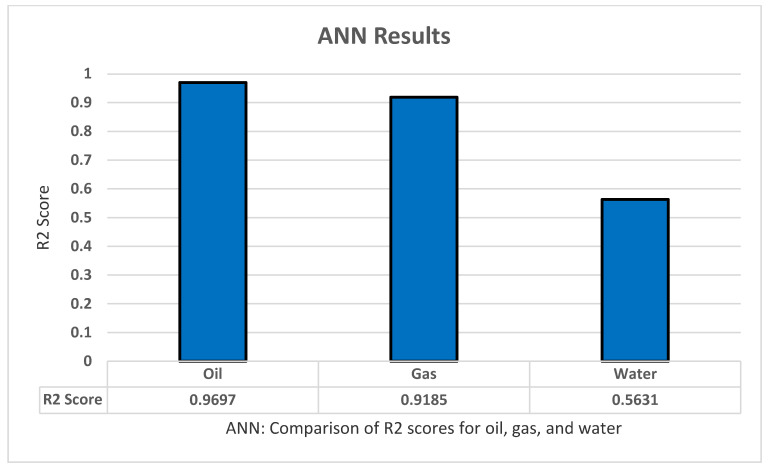
ANN’s R2 score results.

For the ANN model, we began our experiment using a grid search with an abundance of parameters to fit the best combination possible. We used a sequential classifier of three layers into k-fold cross-validation with five splits and three repeats, we increased the number of epochs to 1000 to ensure better results. The set of parameters are: optimizer ‘ReLu’, Activation ‘Adam’, Init_mode ‘Normal’, Epochs ‘1000’, Batch-size ‘10’, and Learn rate ‘0.3’. This collection of parameters appears to give the best results in predicting the oil production through all three years with a great correlation coefficient score of 0.9697, and thus we applied these parameters to the other tests. Using this model to predict all the gas productions across three years has yielded a score of 0.9185, and low results for the water productions with an R2 score of 0.5631. Lastly, we tested how well the model does through all outputs–all productions and ratios–and obtained a good score of 0.8506 compared with the other models as shown in Figure 10.

### 5.8. RNN(LSTM)

**Figure 11:**
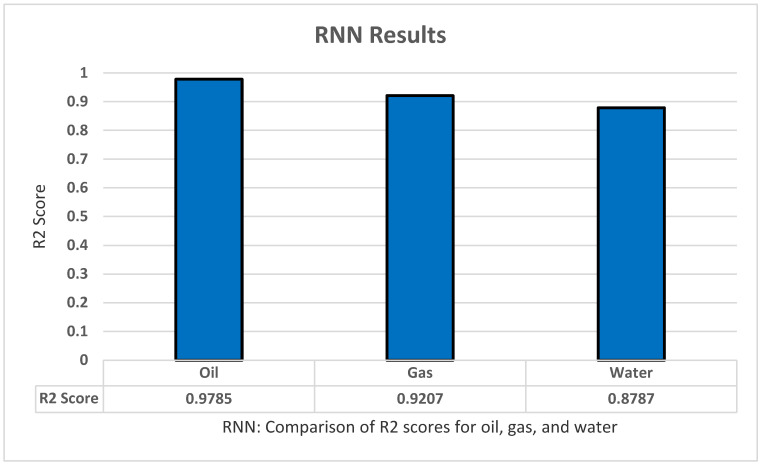
RNN’s R2 score results.

In RNN we used LSTM from the Kkeras library. We started with an LSTM input layer with 104 hidden nodes. Then we added 200 nodes in the hidden layer, a dropout of 0.2, and a dense output layer consisting of 35 nodes representing each month. Based on our tests for ANN we used adam as an optimizer and mean_squared_error for loss. The model was trained with a batch size of 50 and 400 epochs. The model performed well with an R2 score of 0.9785 for oil prediction as shown in Figure 11.

Lastly, we used other evaluation metrics in our ANN model, as its performance is one of the best in our experiments. As shown in Table 3 we can see the results of MAE, MSE, and RMSE.

**Figure 12:**
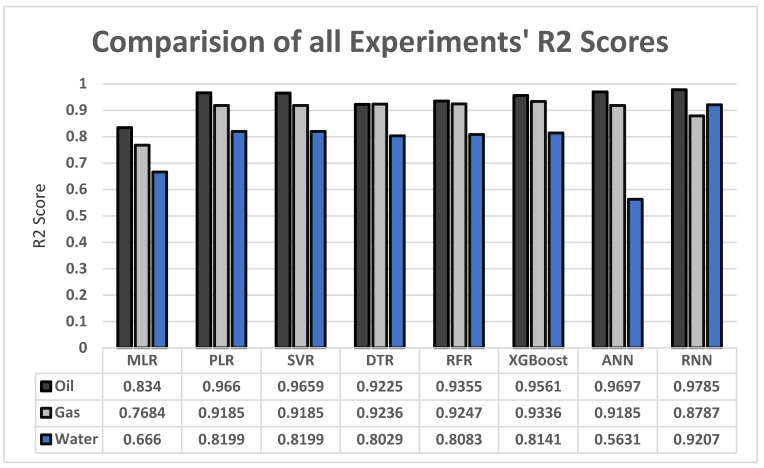
All models’ R2 score results comparision.

The oil results are the best-performing according to the results of our experiments in Figure 12, followed by the gas results. The water results show a small drop compared to oil and gas, which is caused by the nature of the dataset as it is more focused on oil and gas.

### 5.9. Experiments Discussion

The main advantage this paper provides is a fast and accurate prediction of a well’s production and characteristics in comparison with the current simulation methods, which take much more time and require more computational power. However, this is limited by the need for many records of wells or simulations of wells with different properties and geological features to accurately represent future wells.

The main limitation of this paper’s experiments is the dataset. The dataset represents wells in Saudi Arabia and, based on the location, the geological features differ, such as the porosity and permeability of rocks. This limits the application of this research to wells with similar properties. This limitation, however, can be overcome by acquiring datasets that represent different types of reservoirs and their production records and features. Another issue with the dataset is that the water productions are not as consistent as the gas and oil productions which lead to a significant drop in water production prediction compared to oil and gas production predictions. XGBoost and RNN, however, showed us the greatest water results according to Figure 12. We are planning to experiment more on these two models and more similar new methods to overcome the water’s low prediction issue.

## 6. Conclusions

In this paper, we attempted to accelerate the process of predicting oil and gas production using ML and DL methods. The models were passed through a series of transform functions that were applied to the data. Below are the main highlights of our findings:

- The results we achieved with ANN, XGBoost, and RNN are the highest, with a mean R2 for oil, gas, and water of 0.9627, 0.9012, and 0.926, respectively. We found that ML algorithms performed best with the default dataset while the other algorithms performed better in the custom dataset. Some methods had more significant results if the data were standardized before experimenting, such as SVR with a mean R2 of 0.9014. Other algorithms, however, performed better with a pure dataset such as RFR with a mean R2 of 0.8848. Normalizing the dataset for both the default and the custom datasets did not yield good results and was outperformed by pure and standardized data.
- After experimenting with the dataset and examining the results for every method selected, it is hard to say that these are the best results we can obtain. There is still plenty of room for improvement to achieve even better results by exploring different methods or a combination of methods. Nevertheless, the results we acquired are satisfactory considering the complexity of the problem.

## 7. Future Work

The progress and the results that we obtained are not final. We aim to improve and test on different set of methods, and even return to the previous ones and perform further tests to achieve better results. One of the of ideas we want to work toward is creating a system that can select an ML or DL models depending on the dataset type, features, and more.

## Figures and Tables

**Table 1:** Dataset Description.

Column Name	Description
Well location	Each well will have an index that represents the location of the well in the *X* and *Y* axis. The column name for *X* axis is I, and for *Y* is J.
Contact	We have a contact zone for oil, water, and gas. Each column will have a fraction that represents how much the well is in contact with each attribute.
Permeability average	This feature tells us about the average in three directions: *X*, *Y*, and *Z*, of how much the material under the well such as rooks, can transmit fluids.
Volume	The volume is how much of oil, water, and gas is around the well, and it is represented in numeric values.
Production	Each well will have 35 columns for the oil, water, and gas. Every column will represent a value of oil, water, gas production rate for a three-year simulation period.
Wellhead and bottomhole pressure	Both these features will have 35 values over the three-year simulation period. Wellhead pressure is the pressure at the top of the well, and bottom-hole pressure is the pressure at the bottom of the hole of the well.
Ratio	We will have ratios for gas and oil (GOR), gas and water (GWR), and oil and water (OWR).

**Table 2:** All methods parameters optimized.

Method	Parameter	Parameters Value
MLR	Fit_intercept	True
	Positive	True
PLR	Fit_intercept	True
	Positive	True
SVR	Kernel	Rbf
	Gamma	scale
	C	475
	Epsilon	0.01
	Max_iter	−1
	Tol	0.1
DTR	Criterion	‘absolute_error’
	max_depth	6
	max_features	‘auto’
RFR	criterion	‘squared_error’
	n_estemators	100
	max_features	‘auto’
XGBoost	Max_depth	2
	Learning rate	0.4
	Booster	‘gnlinear’
	Gamma	0
ANN	Optimizer	ReLu
	Activation	Adam
	Init_mode	Normal
	Epochs	1000
	Batch-size	10
	Learn rate	0.3
RNN	Optimizer	Adam
	dropout	0.2
	Dense	35
	Epochs	400
	Batch-size	50
	verbose	0

**Table 3:** Other evaluation measures on the ANN model.

	Oil	Gas	Water
MAE	0.1223	0.1563	0.2732
MSE	0.0318	0.0597	0.1798
RMSE	0.1777	0.2212	0.3706
